# The large conductance Ca^2+^ -activated K^+^ (BKCa) channel regulates cell proliferation in SH-SY5Y neuroblastoma cells by activating the staurosporine-sensitive protein kinases

**DOI:** 10.3389/fphys.2014.00476

**Published:** 2014-12-09

**Authors:** Angela Curci, Antonietta Mele, Giulia Maria Camerino, Maria Maddalena Dinardo, Domenico Tricarico

**Affiliations:** Department of Pharmacy-Drug Science, University of Bari “Aldo Moro”Bari, Italy

**Keywords:** maxi-calcium activated K^+^-channels, cell proliferation, voltage dependent K^+^-channels, SH-SY5Y neuroblastoma cells, protein kinases, staurosporine

## Abstract

Here we investigated on the role of the calcium activated K^+^-channels(BKCa) on the regulation of the neuronal viability. Recordings of the K^+^-channel current were performed using patch-clamp technique in human neuroblastoma cells (SH-SY5Y) in parallel with measurements of the cell viability in the absence or presence of the BKCa channel blockers iberiotoxin(IbTX) and tetraethylammonium (TEA) and the BKCa channel opener NS1619. Protein kinase C/A (PKC, PKA) activities in the cell lysate were investigated in the presence/absence of drugs. The whole-cell K^+^-current showed a slope conductance calculated at negative membrane potentials of 126.3 pS and 1.717 nS(*n* = 46) following depolarization. The intercept of the I/V curve was −33 mV. IbTX(10^−8^ – 4 × 10^−7^ M) reduced the K^+^-current at +30 mV with an IC_50_ of 1.85 × 10^−7^ M and an Imax of −46% (slope = 2.198) (*n* = 21). NS1619(10–100 × 10^−6^ M) enhanced the K^+^-current of +141% (*n* = 6), at −10 mV(Vm). TEA(10^−5^–10^−3^ M) reduced the K^+^-current with an IC_50_ of 3.54 × 10^−5^ M and an Imax of −90% (slope = 0.95) (*n* = 5). A concentration-dependent increase of cell proliferation was observed with TEA showing a maximal proliferative effect(MPE) of +38% (10^−4^ M). IbTX showed an MPE of +42% at 10^−8^ M concentration, reducing it at higher concentrations. The MPE of the NS1619(100 × 10^−6^ M) was +42%. The PKC inhibitor staurosporine (0.2–2 × 10^−6^ M) antagonized the proliferative actions of IbTX and TEA. IbTX (10 × 10^−9^ M), TEA (100 × 10^−6^ M), and the NS1619 significantly enhanced the PKC and PKA activities in the cell lysate with respect to the controls. These results suggest that BKCa channel regulates proliferation of the SH-SY5Y cells through PKC and PKA protein kinases.

## Introduction

The calcium activated K^+^-channels (BKCa) channels are ubiquitously present in most human cells and play an essential role in the regulation of basic cellular processes such as electrical excitability of cell membrane, vascular tone, neurotransmitter release (Zhang et al., 2003; Tricarico et al., 2005; Lee and Cui, 2010).

BKCa channels are composed by the alpha subunit encoded by one gene (slo1/KCNMA1) assembled as tetramer and transmembrane beta subunits (beta1-4) encoded by KCNMB1-4 genes. The alpha subunit of BKCa channels may assemble with beta-subunits with 1:1 stoichiometry enhancing the calcium sensitivity, favoring the trafficking into the membrane and modulating the pharmacological responses (Kyle and Braun, 2014). The alpha, alpha+beta 1, alpha+beta 2/3, or beta 4 mimics the skeletal muscle, vascular smooth muscle and neuronal BKCa channels, respectively (Orio and Latorre, 2005; Lee and Cui, 2010). Furthermore, splicing isoforms of the alpha subunit gene are expressed in the tissues including skeletal muscle affecting physiological properties and pharmacological response of the native channels (Dinardo et al., 2012). More recently gamma subunits have been described (Toro et al., 2014). The gamma subunits are auxiliary leucine-rich repeat (LRR) -containing protein 26 (LRRC26) that following interaction with the BKCa channel lead to channel activation at negative voltages without rising in the intracellular calcium concentration. Several gamma subunits have been identified in excitable and non-excitable tissues modulating the gating of a BKCa channel by enhancing the allosteric coupling between voltage-sensor activation and the channel's closed-open transition (Yan and Aldrich, 2010, 2012).

Activation of BKCa channels has been reported to be involved in the regulation of cell viability and apoptosis besides its electrophysiological function. Emerging evidences suggest that the BKCa channel plays a role in cell viability in different cell types including osteoblasts, vascular smooth muscle cells and in cell lines expressing the recombinant channel subunits (Henney et al., 2009; Jia et al., 2013). It has been recently shown that high glucose enhances HEK293 cell viability by inhibition of cloned BKCa channel subunits hslo + beta 1 (Chang et al., 2011). The BKCa channel openers NS1619 or tamoxifen significantly induced apoptosis reducing cell viability in cells expressing the combination of the hslo + beta 1 subunits under hyperglycemia conditions indicating that cloned BKCa channel regulates apoptosis and proliferation of HEK293 cell. These findings suggest that this effect may have a role in the diabetic vascular dysfunction associated with a vascular wall hypertrophy (Chang et al., 2011). In line with this observation, we recently demonstrated that the hslo subunit regulates the cell viability in response to changes of the external K^+^ ion concentration (Tricarico et al., 2013). The cell viability after 24 h of incubation under hyperkalemia was enhanced by 82 ± 6 and 33 ± 7% in hslo-HEK293 cells and HEK293 cells, respectively. The BKCa channel blockers iberiotoxin (IbTx), charybdotoxin (ChTX), and tetraethylammonium (TEA) enhanced cell viability in hslo-HEK293 and the BKCa openers prevented the enhancement of the cell viability induced by hyperkalemia or IbTx in hslo-HEK293. In contrast, under hypokalemia cell viability was reduced by −22 ± 4 and −23 ± 6% in hslo-HEK293 and HEK293 cells, respectively, thereby suggesting that the BKCa channel regulates cell viability under hyperkalemia but not hypokalemia conditions. These findings may have relevance in the neuromuscular disorders associated with abnormal K^+^ ion homeostasis including periodic paralysis and myotonia. Hyperkalemia condition associated with hypertrophic phenotype is indeed often observed in myotonia (Adrian and Bryant, 1974; Cannon, 1996).

The role of BKCa channels in the proliferation process in the tumor cells is controversial. Some studies have suggested that BKCa channels contributed to the high proliferative or invasive potential in a number of malignant cell lines, such as non-metastatic (MCF-7) breast cancer cells (Ouadid-Ahidouch and Ahidouch, 2008), brain-specific metastatic (MDA-MB-361) breast cancer cells (Khaitan et al., 2009), human prostate cancer (Bloch et al., 2007), colorectal carcinogenesis (Koehl et al., 2010), and glioma (Weaver et al., 2006; Sontheimer, 2008).

Some others concluded that BKCa channels are not required for the proliferation in glioma (Abdullaev et al., 2010) or breast cancer cells because the BKCa channel blockers charybdotoxin or iberiotoxin did not affect cell proliferation (Roger et al., 2004).

In contrast, BKCa channels have been reported to exhibit anti-proliferative and anti-tumorogenic properties in osteosarcoma cells, ovarian cancer cells, glioma cells and in human MDA-MB-231 breast cancer cells. The bisphosonates Zoledronic acid used in the osteoporosis associated with bone metastasis and the BKCa channel opener NS1619, induced apoptosis through the opening of BKCa channels, while hslo gene silencing or channel blockers induced cell proliferation (Cambien et al., 2008; Han et al., 2008; Debska-Vielhaber et al., 2009; Ma et al., 2012).

We therefore investigated on the role of BKCa channels in the proliferation process in SH-SY5Y neuroblastoma cells. This cell type shows a high activity of BKCa channels, but their specific contribution to the proliferation process is not known (Park et al., 2010).

Moreover, very little is known about the non-conducting functions of BKCa channels, in particular which signaling cascades they modify. In this work, the ion channel characterizations were performed using patch–clamp techniques in SH-SY5Y neuroblastoma cells. Cell proliferation was investigated by evaluating the mitochondrial succinic dehydrogenases activity, cell diameter and volume changes. The capability of the staurosporine (STS), a well-known not selective protein kinase C inhibitor, to antagonize the drug-induced cell proliferation was also investigated. The involvement of the protein kinase C (PKC) and protein kinase A (PKA) in the drug-induced cell proliferation was investigated using an enzyme-linked immuno-absorbent assay (ELISA) assay in the cell lysates.

Our findings may have relevance either in the neuromuscular disorders where this mechanism may play a role in the cell repair and in the proliferative disorders. PKC and PKA enzymes other than recognized pathways involved in the cell proliferation may have a role in the repair processes.

## Materials and methods

### Drugs and solutions

In whole cell patch-clamp experiments, the pipette (intracellular) solutions contained (10^−3^ M): 132 K^+^-glutamate, 1 ethylene glycol bis (β-aminoethyl ether)-N, N, N, N-tetraacetic acid (EGTA), 10 NaCl, 2 MgCl_2_, 10 HEPES, 1 Na_2_ATP, 0.3 Na_2_GTP, pH = 7.2 with KOH. The bath solution contained (10^−3^ M): 142 NaCl, 2.8 KCl, 1 CaCl_2_, 1 MgCl_2_, 11 glucose, 10 HEPES, pH = 7.4 with NaOH. CaCl_2_ was added to the pipette solutions to give free Ca^2+^ ion concentration of 1.6 × 10^−6^ M in whole cell experiments. The calculation of the free Ca^2+^ ion concentration in the pipette was performed using the Maxchelator software (Stanford University, USA).

The BKCA opener under investigation was NS1619. The selective and impermeant BKCA blocker investigated here was iberiotoxin (IbTX), the unselective blocker was tetraethylammonium (TEA). The Kir blocker used was Ba^2+^ ions. The drugs and toxins were purchased from Sigma (SIGMA Chemical Co., Mi, Italy). Stock solutions of the drugs under investigation were prepared dissolving the drugs in dimethylsulphoxide (DMSO) at concentrations of 118.6 × 10^−3^ M. Microliter amounts of the stock solutions were then added to the bath solutions as needed. DMSO did not exceed 0.07% in the bath, at this concentration the solvent does not normally affects the K^+^-current or cell proliferation. Cell viability experiments were performed in Dulbecco's Modified Eagle's Medium (DMEM) (+) solution enriched with 1X antibiotics (1%), L-glutamine (1%), and fetal serum albumin (FBS) (10%).

### Patch-clamp experiments

The K^+^-currents and drug actions on the channel currents were investigated in the human neuroblastoma cell line SH-SY5Y during voltage steps, in the range of potentials going from −150 mV(Vm) to +110/+150 mV (Vm), *HP* = −60 mV (Vm), in the presence of internal Ca^2+^ ions, in asymmetrical K^+^ ion concentrations (int K^+^: 132 × 10^−3^ M; ext K^+^: 2.8 × 10^−3^ M) using whole-cell patch-clamp technique. The resulting K^+^-current was a leak subtracted and normalized to capacitance. Drug effects were investigated in a physiological range of potentials from −10 mV (Vm) to + 30 mV (Vm) for all drugs. The K^+^-current was recorded at 20°C and sampled at 1 kHz (filter = 2 kHz) using an Axopatch-1D amplifier equipped with a CV-4 headstage (Axon Instruments, Foster City, CA). The channel currents were identified on the basis of their voltage dependence and response to toxins and drugs. The leak currents were measured in the presence of saturating concentration of Ba^2+^ (5 × 10^−3^ M) and TEA (5 × 10^−3^ M) which caused a full block of Kir, Kv, and BKCA channels.

Current analysis was performed using pClamp 10 software package (Axon Instruments). The criteria for accepting the data entering were based on the stability of the seal evaluated by observing the noise levels not exceeding 0.6 pA at 2 kHz. Pipettes resistance was 9 ± 0.2 MΩ (Number of pipettes = 150).

The cells were exposed to the drug solutions for 2 min. before recordings. Increasing concentrations of drug solutions were applied to the cells by the fast perfusion system (AutoMate, Sci. Berkeley, California 94710, USA). Each application of drug solution was followed by a washout period of 1 min to allow recovering of channel currents to control values. No more than three different drug concentrations were applied to the same cell, with one compound per cell tested at a time. Due to the not reversibility of the IbTX action following washout during the time of observation, only one concentration per cell and plate was tested at a time for this drug. Seal resistance was continuously monitored during patch solutions exchange.

### Cell viability: mitochondrial succinic dehydrogenases activity assay

Cell viability was evaluated by measuring the succinic dehydrogenases activity in the cell suspension using the cell counting Kit-8 (CCK-8) (Enzo Life Sciences International, Inc., USA) which utilizes highly water-soluble tetrazolium salt. WST-8 2- (2- methoxy -4-nitrophenyl) -3-(4- nitrophenyl)- 5-(2,4- disulfophenyl)-2H- tetrazolium, monosodium salt produces a water-soluble formazan dye upon reduction in the presence of an electron carrier. It is reduced by mitochondrial dehydrogenases in cells to give a yellow colored product (formazan), which is soluble in the tissue culture medium. The detection sensitivity of CCK-8 is higher than other tetrazolium salts. The changes of the cell vitality were expressed as % changes of cell viability induced by drugs and toxin with respect to the controls.

### Cell viability: cell volume assay

Measures of cell volume were based on the relationship existing between voltage changes and cell volume changes. Precise cell volumes are drawn into a sensor and the measurements are based on the impedentiometric principle. As cells flow through the aperture in the sensor, resistance increases. This increase in resistance causes a subsequent increase in voltage. Voltage changes are recorded as spikes with each passing cell and it is proportional to the cell volume. The spikes of the same size are bucketed into a histogram and counted. This histogram gives the quantitative data on cell morphology that can be used to examine the quality and health of your cell culture. The Scepter™ 2.0 cell counter (MERK-Millipore, USA) is compatible with 60 and 40 μm sensors, in our experiments we used the 60 μm sensor for particles between 6 and 36 μm.

### Protein kinase activity assay

The PKA and PKC activities assay used in our experiments are based on a solid phase ELISA that utilizes a specific synthetic peptide as a substrate for PKA or PKC and a polyclonal antibody that recognizes the phosphorylated form of the substrate (Abcam, Cambridge, UK). The assay is designed for analysis of PKA or PKC activity in the solution phase. For statistical results, the assays were run in triplicate. The data from the crude protein preparations were compared with the data obtained from the standard calibration curves performed using the purified enzymes.

### Data analysis and statistics

The data were collected and analyzed using Excel software (Microsoft Office 2010). The data are expressed as mean ± S.E. unless otherwise specified.

In the case of the channel blockers IbTX and TEA molecules and Ba^2+^ ions the data could be fitted with the following equation:

$$\begin{array}{l} (I\text{drug}-I\text{control}/I\text{control}-I\text{max})\;\times \;100\\ \;=(1+\text{IC50/}[\text{Drug}])n/I\text{max} \end{array}$$

*I* drug is the K^+^-current measured in the presence of the molecules under study and normalized to the maximal currents recorded in the same patches; IC50 is the concentrations of the drugs needed to inhibit the current by 50%; Drug is the concentration of the drug tested; *I* max is the maximal current recorded in the patches at 110/150 mV (Vm); *I* control is the current recorded in the absence of drugs; and *n* is the slope factor of the curve. The algorithms of the fitting procedures used are based on a Marquardt least-squares fitting routine. Data analysis and plot were performed using SigmaPlot software (Systat Software, Inc., San Jose, CA).

The % change of the cell viability induced by the drugs and toxin, was calculated in respect to the controls (absence of blockers) using the following equations:

%change of the cell viability=([drugs]/Controls)×100.

The Scepter™ Software Pro was used for the calculation of the cell diameter and volume in the cell population (MERK-Millipore, USA). Data can be exported and further analyzed using excel software (Microsoft Office 2010).

The % activation of kinases activity (Relative PKC or PKA activity drug/Relative PKC activity CTRL) × 100.

The % inhibition of kinases activity (Relative PKC activity drug/Relative PKC activity CTRL) × 100 – 100.

Differences between mean were evaluated using the student *t*-test, at *p* < 0.05 level of significance.

## Results

**Figure 1:**
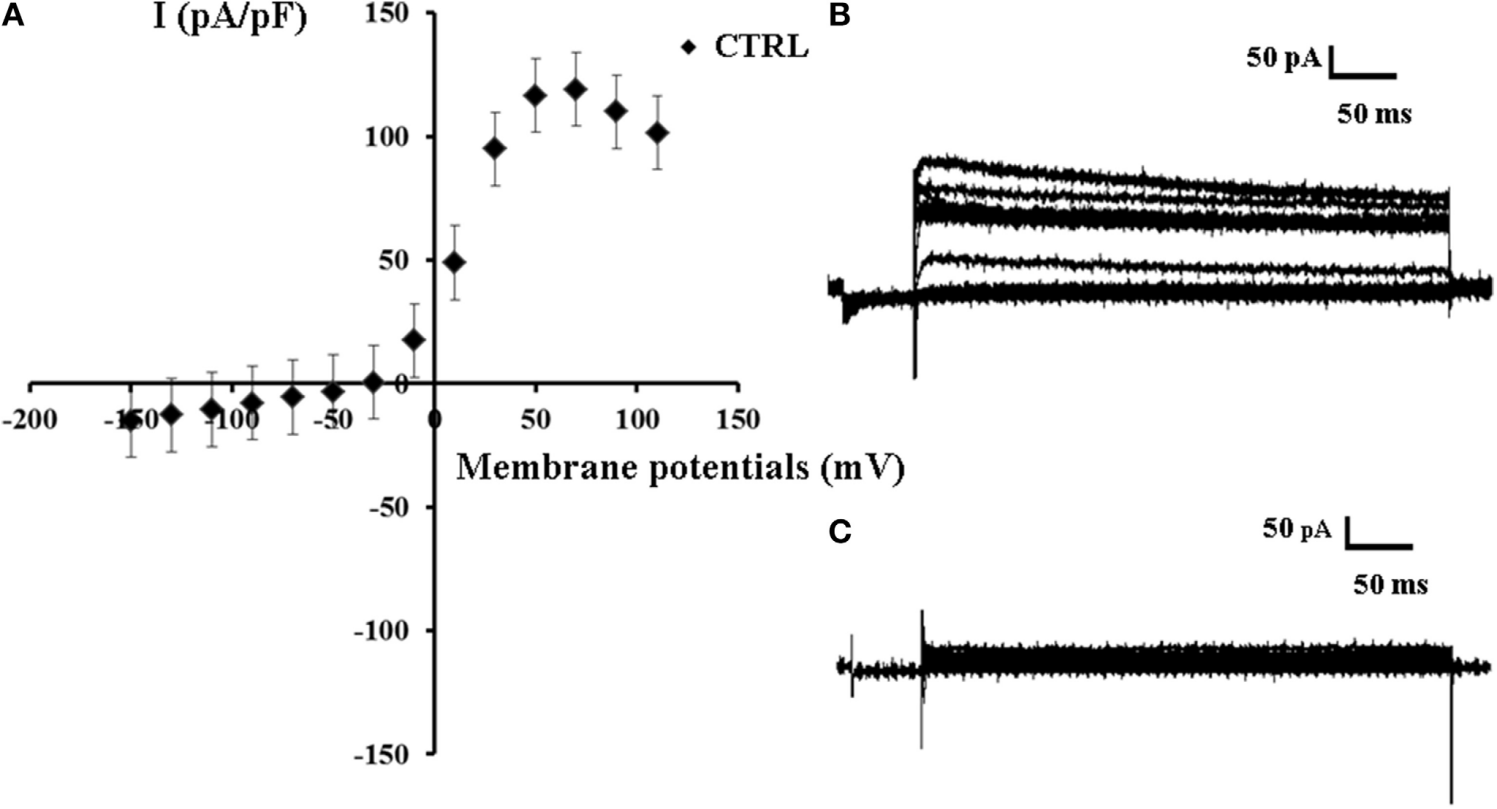
**Current voltage-relationship of K^+^ -current recorded in SH-SY5Y neuronal cell line. (A)** A sigmoid I/V relationship of the mean K^+^ ions currents (*n* = 46) recorded in the control condition (CTRL) in asymmetrical K^+^ ions concentrations (int K^+^: 132 × 10^−3^ M; ext K^+^: 2.8 × 10^−3^ M), in the presence of internal 1.6 × 10^−6^ M concentration of free Ca^2+^ ions, in the range of potentials going from −150 to +110 mV, *HP* = −60 mV (Vm), using whole-cell patch-clamp technique. The K^+^-current was leak subtracted and normalized to capacitance. The intercept of the I/V curve on the voltage membrane axis was −33 mV. **(B)** Sample traces of the K^+^ ions current recorded in the control conditions. **(C)** Sample trace of the leak current recorded in asymmetrical K^+^ ion concentrations in the presence of Ba^2+^ ions (5 × 10^−3^ M) and TEA (5 × 10^−3^ M) in the bath solutions.

In our experiments the whole cell K^+^-current recorded in asymmetrical K^+^ ion concentrations (int K^+^: 132 × 10^3^ M; ext K^+^: 2.8 × 10^−3^ M) and internal free Ca^2+^ ions of 1.6 × 10^−6^ M concentration showed a sigmoid I/V relationship in the range of membrane potentials from −150 to +70 mV; a decay of the current was observed at membrane potentials >+70 mV which is consistent with the presence of the inactivation process. The slope conductance calculated in the range of membrane potentials from −150 to −30 mV was 126.3 ± 11 pS, and it was 1.717 ± 101 nS (*n* = 46) in the range of membrane potentials from −10 to +50 mV (Figures 1A–C). The intercept of the I/V curve on the voltage membrane axis was −33 mV which is consistent with the depolarized resting potential characterizing the SH-SY5Y neuronal cell line (Yang and Brackenbury, 2013).

**Figure 2:**
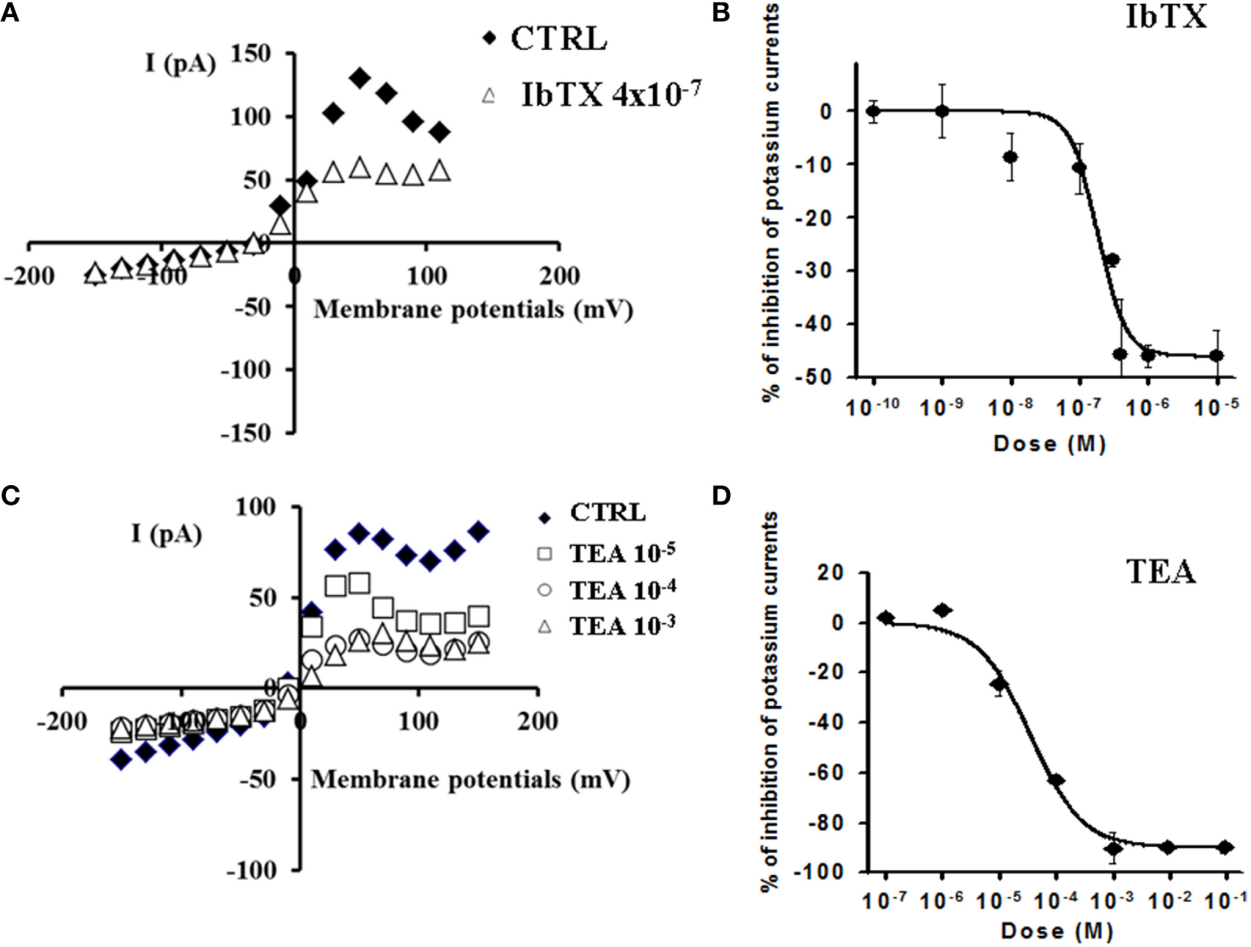
**Effects of the BKCa channel blockers IbTX and TEA on K^+^-current recorded in SH-SY5Y neuronal cell line**. The effects of IbTX and TEA were investigated on the K^+^-current recorded in asymmetrical K^+^ ions concentrations (int K^+^: 132 × 10^−3^ M; ext K^+^: 2.8 × 10^−3^ M), in the presence of internal 1.6 × 10^−6^ M concentration of free Ca^2+^ ions, in the range of potentials going from −150 to +110 mV, *HP* = −60 mV using whole cell patch clamp technique. The whole cell K^+^-current was a leak subtracted and normalized to capacitance. **(A)** I/V relationship in the absence or presence of IbTX from a single patch. IbTX (4 × 10^−7^ M) reduced the outward K^+^-current of the −53% at +30 mV (Vm) in this patch. **(B)** IbTX (10^−10^–10^−5^ M) induced a concentration-dependent reduction of the K^+^-current at +30 mV (Vm). **(C)** I/V relationship in the absence or presence of increasing concentrations of unselective K^+^ channel blocker TEA from a single patch. **(D)** TEA (10^−5^–10^−3^ M) induced a concentration-dependent reduction of the outward K^+^- current. A full reduction of the K^+^-current on −100% at +30 mV (Vm) was observed in the presence of TEA at 10^−3^ M concentration.

The concentration-response relationships were investigated at −10, +10, and +30 mV of voltage membrane which are physiological membrane potentials for this cell line at which BKCA channel should be operative. The application of the BKCa channel blocker IbTX (4 × 10^−7^ M) induced a significant reduction of the outward K^+^-current on −53% at +30 mV (Vm) which was not-reversible following washout of the toxin solution (Figure 2A). IbTX (10^−10^ – 4 × 10^−5^ M) induced a concentration-dependent reduction of the K^+^-current with an IC_50_ of 1.85 × 10^−7^ M and an Imax of −46% (slope = 2.198) at +30 mV(Vm) (*n* = 21) (Figure 2B).

The application of the unselective K^+^ channel blocker TEA (10^−5^–10^−3^ M) induced a concentration-dependent reduction of the outward K^+^- current which was reversible following washout of the drug solution. A full reduction of the K^+^-current on −100% at +30 mV (Vm) was observed in the presence of TEA at 10^−3^ M concentration (Figure 2C). Concentration-response analysis showed that TEA (10^−7^–10^−1^ M) reduced the K^+^-current with an IC_50_ of 3.54 × 10^−5^ M and an Imax of −90% (slope = 0.95) (*n* = 5) (Figure 2D). No significant effects of IbTX and TEA were observed on the K^+^-current at negative membrane potentials.

**Figure 3:**
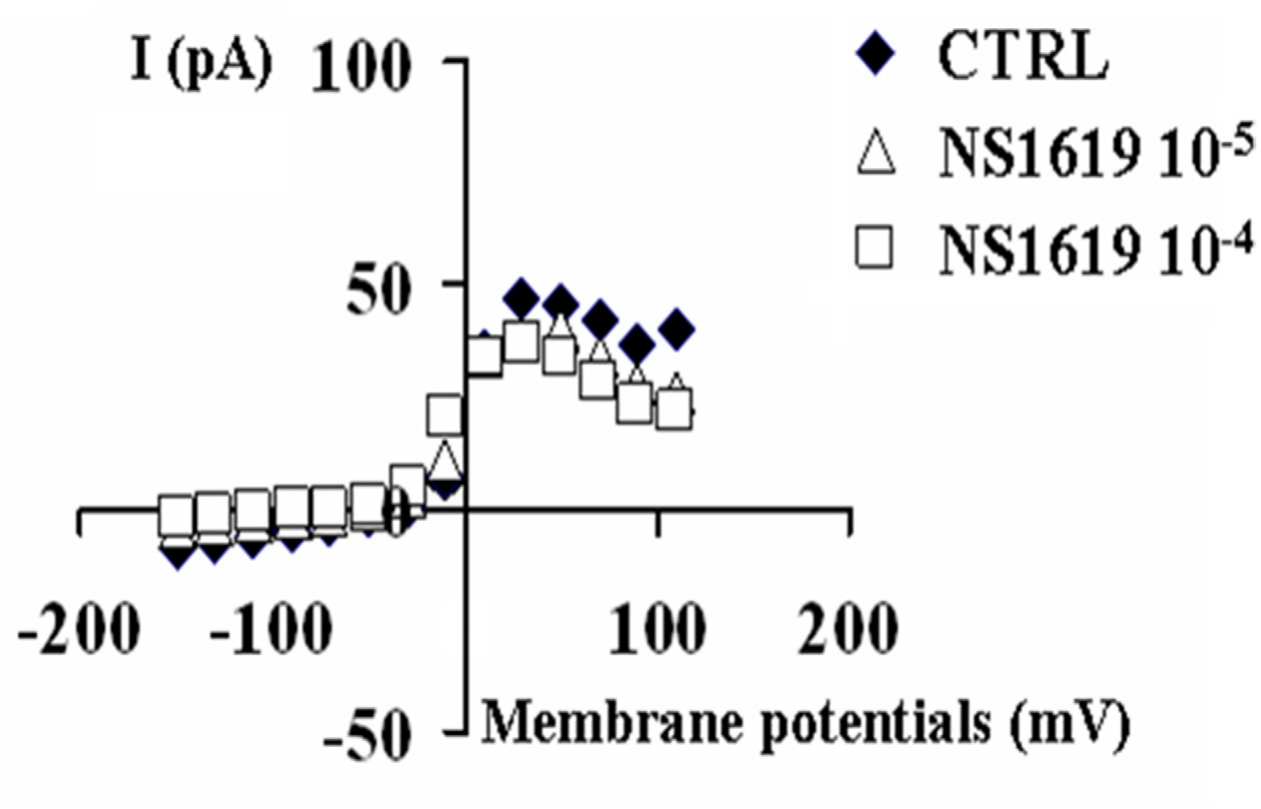
**Effects of the BKCa channel opener NS1619 on K^+^-current recorded in SH-SY5Y neuronal cell line**. The effects of the NS1619 (10 × 10^−6^ M and 100 × 10^−6^ M) were investigated on the K^+^-current recorded in asymmetrical K^+^ ions concentrations (int K^+^: 132 × 10^−3^ M; ext K^+^: 2.8 × 10^−3^ M), in the presence of internal 1.6 × 10^−6^ M concentration of free Ca^2+^ ions, in the range of potentials going from −150 to +110 mV, *HP* = −60 mV using whole cell patch clamp technique. The K^+^-current was leak subtracted and normalized to capacitance. The I/V relationship was constructed in the absence (CTRL) or presence of different concentrations of NS1619. In the this experiment, the application of 10 × 10^−6^ M and 100 × 10^−6^ M concentrations of this drug enhanced the K^+^-channel current, at −10 mV (Vm), respectively by +41.7 and +171% with respect to the control.

The BKCa channel opener NS1619(10–100 × 10^−6^ M) enhanced the K^+^-current in the range of potentials from −10 mV to +10 mV. This drug enhanced the K^+^-current of +41.7% (*n* = 6) and +141% (*n* = 6) respectively at 10 × 10^−6^ M and 100 × 10^−6^ M, at −10 mV(Vm), while leading to the a mild reduction of the K^+^-current of −23.15% at +30 mV(Vm) suggesting a possible interaction of this drug with Kv channels other than BKCa channels (Figure 3).

**Figure 4:**
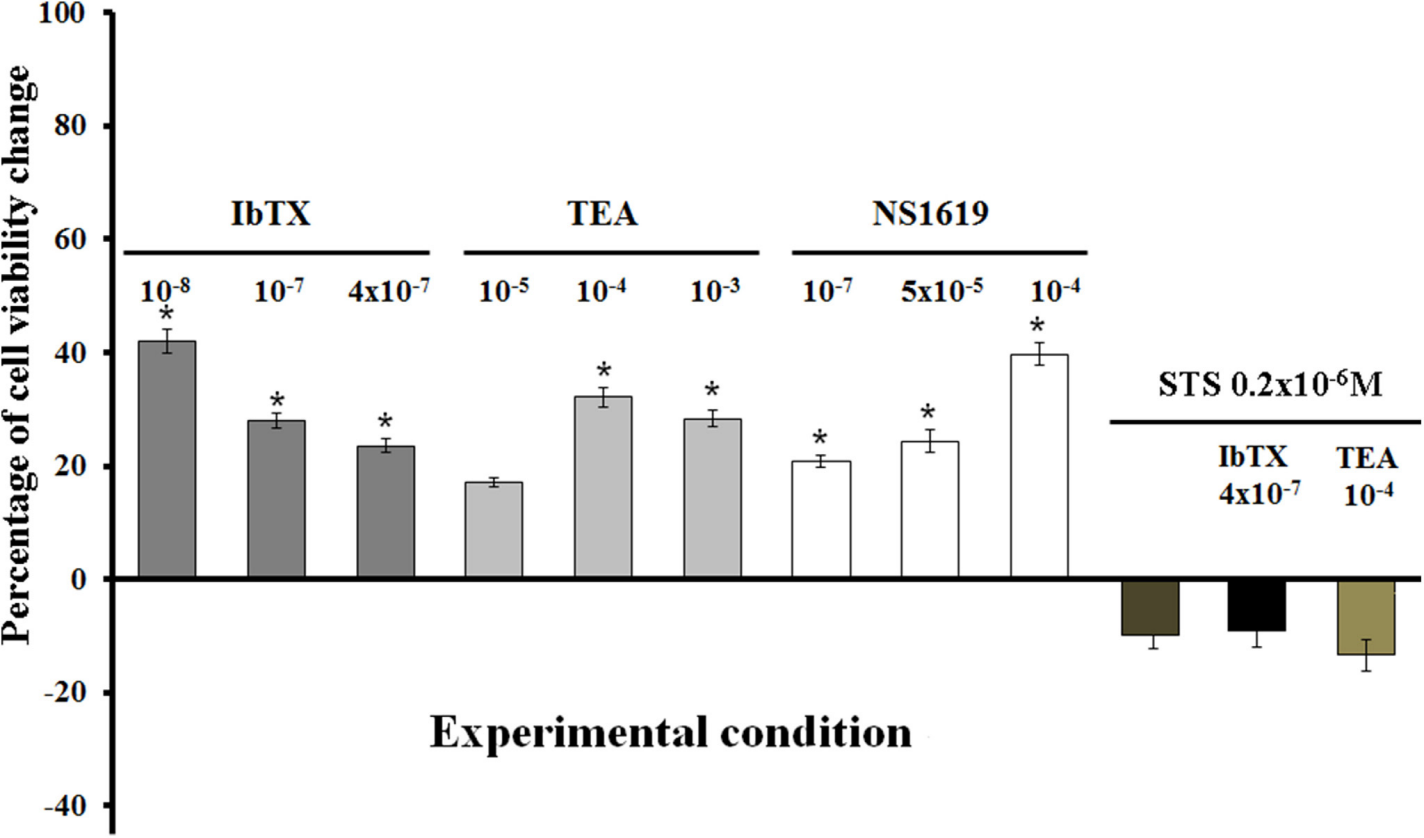
**Effects of the channel blockers IbTX and TEA and BKCa channel opener NS1619 on the viability of SH-SY5Y cells**. Concentration-dependent increase of the cell proliferation observed with TEA as determined by mitochondrial succinic dehydrogenase activity assay. An inverse relationship was observed with IbTX showing a maximal proliferative effect on 10^−8^ M concentration, but reducing cell proliferation at high concentrations. The NS1619 enhanced cell proliferation showing a maximal proliferative effect of +117%. The co-incubation of the cells with IbTX/TEA+STS(2 × 10^−6^ M) fully antagonized the proliferative actions of the IbTX and TEA, also leading to cell death in respect to the controls. ^*^Data significantly different with respect to the controls for *p* < 0.05 as determined by student *t*-test.

A concentration-dependent increase of the cell proliferation was observed following 6 h of incubation time of the cells in the presence of TEA showing a maximal proliferative effect (MPE) of +38% at 10^−4^ M concentration as determined by mitochondrial succinic dehydrogenase activity assay. IbTX caused an MPE of +42% at a 10^−8^ M concentration, but reducing its efficacy at higher concentrations (Figure 4). This may be related to the fact that the toxin at higher concentration *per se* may lead to unspecific actions on cell viability unrelated to the BKCa channel blocking mechanism.

The BKCa channel opener NS1619 also induced cell proliferation showing an MPE of +42% at 10^−4^ M concentration. The co-incubation of the cells with NS1619+IbTx or TEA failed to prevent the enhancement of the cell proliferation induced by IbTX or TEA.

The co-incubation of the cells for 6 h with IbTX or TEA + STS (0.2 × 10^−6^ M) fully antagonized the proliferative actions of the IbTX and TEA (Figure 4). The STS at a 0.2 × 10^−6^ M concentration did not significantly affect the cell viability, while at a 2 × 10^−6^ M concentration, reduced the cell viability causing proteolysis with respect to the controls according to its apoptotic action.

**Figure 5:**
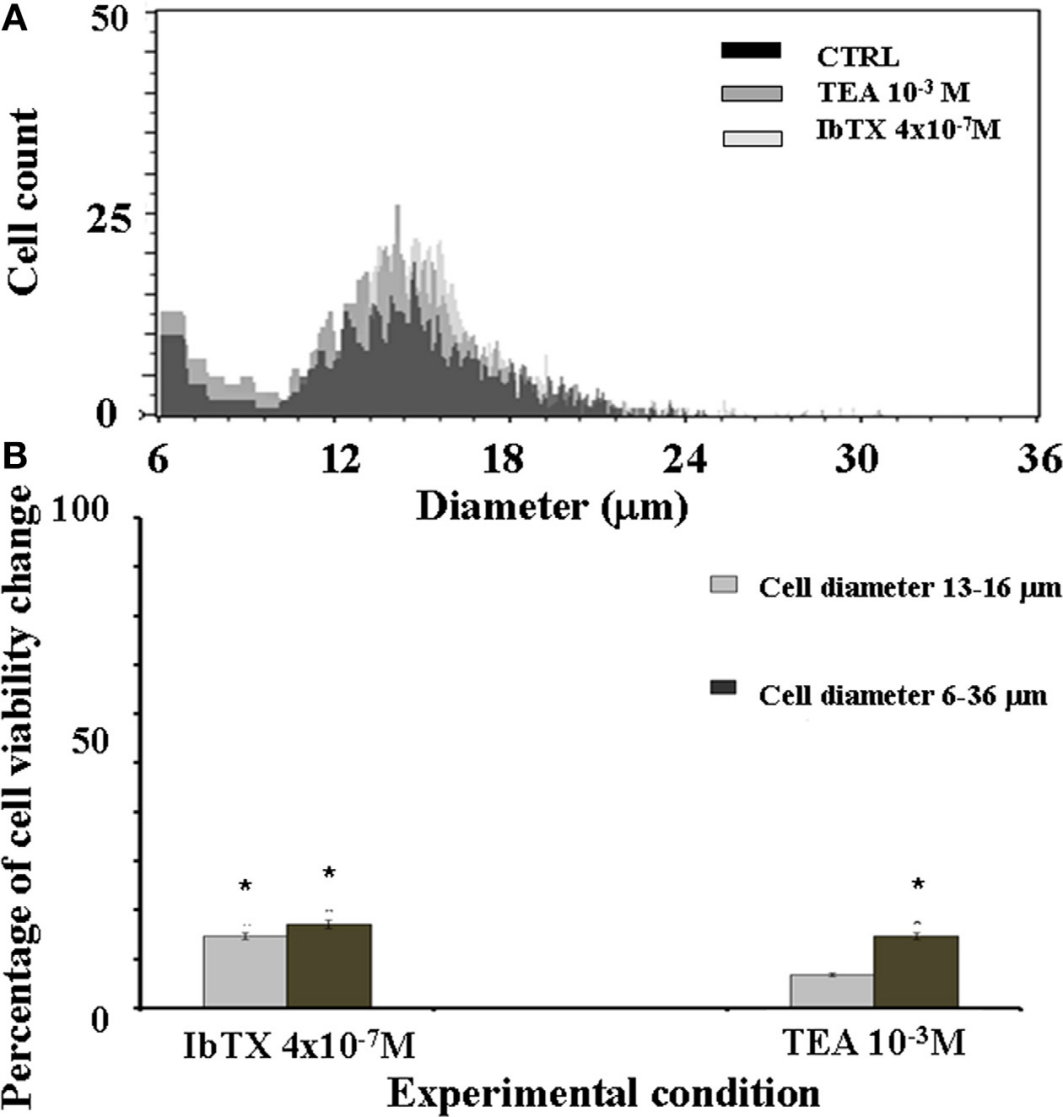
**SH-SY5Y neuronal cell distribution in the presence or absence of the BKCa channel blockers IbTX and TEA**. The effects of the IbTX and TEA on the cell volume were investigated. The cellular distribution followed a Gaussian type distribution **(A)** The most frequently observed cell population in our experimental condition showed a diameter size in the range of 13–16 μm as determined by cell volume assay. The mean cell diameter did not significantly differ between treatments, the values were 14.89 ± 1 in the controls (CTRL) and were 15.02 ± 2 and 14.5 ± 1, respectively, in the cells treated with IbTX and TEA **(B)** After 6 h of incubation time the number of the cells showing a diameter size in the range of 13–16 μm was significantly enhanced by IbTX and TEA. ^*^Data significantly different with respect to the controls for *p* < 0.05 as determined by student *t*-test.

The effects of the IbTX and TEA on the cell volume were also investigated. Cell volume changes are indeed more strikingly related to surface ion channel activity. In our experimental conditions the most frequently observed cell population showed a diameter size in the range of 13–16 μm (Figure 5A). We found that after 6 h of incubation time the number of cells showing a diameter size in the range of 13–16 μm was significantly enhanced by IbTX and TEA (Figure 5B). IbTX (4 × 10^−7^ M) equally enhanced the number of cells showing a diameter size in the range of 6–36 μm suggesting that the observed proliferation is mostly due to an increased number of cells with normal morphology (Figure 5B). IbTX at 4 × 10^−7^ M concentration induced at comparable values of cell proliferation of +22 and +18% (diam. range: 13–16 μm) as determined by the mitochondrial succinic dehydrogenase and the cell volume assays, respectively, suggesting that the proliferative effect of this drug is mediated by a common mechanism affecting either cell volume and the mitochondrial succinic dehydrogenase enzyme. TEA instead caused a significant enhancement of the number of cells showing a diameter size in the range of 6–36 μm in respect to that of the control cells (diam. range: 13–16 μm). This suggests the presence of an abnormal cell population following TEA treatment with a diameter size different from control cells. Moreover, TEA at 10^−3^ M concentration induced a different quantitative enhancement of the cell proliferation of +30 and +19% (diam.: range: 6–36 μm) as determined by the mitochondrial succinic dehydrogenase and the cell volume assays, respectively, suggesting that the two mechanisms may be unrelated to this drug.

**Figure 6:**
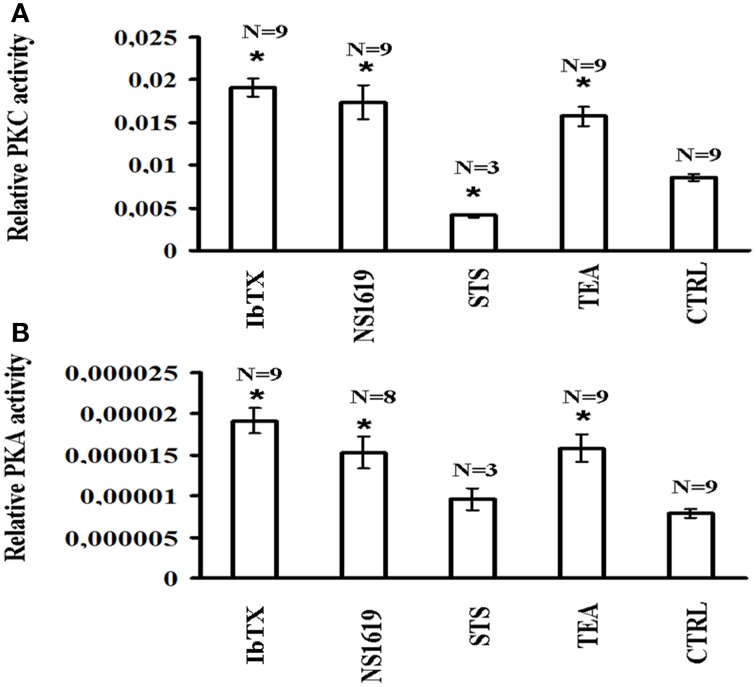
**PKC and PKA activities in SH-SY5Y neuronal cell after 6 h incubation in the presence of the channel blockers IbTX and TEA and opener NS1619**. The effects of the BKCa channel modulators on the protein kinase C (PKC) and protein kinase A (PKA) were investigated in the cell lysate. The cells were incubated with the drugs under investigation for 6 h and the cell lysate were analyzed using an ELISA assay for PKC and PKA activities **(A)** IbTX (10 × 10^−9^ M), TEA (100 × 10^−6^ M), and NS1619 (50 × 10^−6^ M) significantly enhanced PKC activity, while the STS (0.2 × 10^−6^ M) reduced it with respect to the controls **(B)** TEA, IbTX, and NS1819 enhanced PKA activity, while STS was without effect with respect to the controls. ^*^Data significantly different with respect to the controls for *p* < 0.05 as determined by student *t*-test.

The basal PKC activity measured by ELISA assay after 6 h of incubation time was higher than PKA activity in the cells lysate in the control condition. The BKCA channel blockers IbTX (10 × 10^−9^ M) and TEA (100 × 10^−6^ M) enhanced the PKC activity, respectively, by 224.12 and 184.31%, with respect to the controls; the BKCa channel opener NS1619 (50 × 10^−6^ M) also enhanced PKC activity by 203.53% while as expected the STS (0.2 × 10^−6^ M) reduced it by −52.12% (Figure 6A).

The BKCa channel blockers IbTX and TEA also enhanced the PKA activity, respectively, by 241.75 and 199.96% with respect to the controls; the BKCA channel opener NS1619 enhanced PKA activity by 193.23%, while STS did not affect it (Figure 6B).

## Discussion

In the present work we investigated on the role of the BKCa channels in the cell proliferation of the human neuroblastoma cell line SH-SY5Y. In asymmetrical K^+^ ions concentrations, these cells show an elevated K^+^-currents sustained by Kv and BKCa channels, with a minor contribution of the Kirs currents to the total K^+^-currents. The I/V relationship showed an S shaped form going from −150 to +100 mV (Vm) with a reduction of the current amplitude at voltages >100 mV possibly related to inactivation processes characterizing Kv channels. As expected, these cells were depolarized as compared to the native excitable cells such as muscle fibers or neurons (Yang and Brackenbury, 2013; Urrego et al., 2014). In our experiments, the BKCA channels contributed significantly to the total voltage dependent current component; indeed the IbTX sensitive BKCa channel current was about 50% of the total currents recorded following depolarization in the presence of 1.6 × 10^−6^ M concentrations of internal free Ca^2+^ ions.

The BKCa channel blocker IbTX and the unselective K^+^ channel blocker TEA induced a maximal cell proliferation of about 40%, suggesting that BKCa channel and Kv channels similarly contribute to cell proliferation in this cell line. The toxin at a higher concentration of 4 × 10^−7^ markedly reduced channel currents of −47%, enhanced cell proliferation by +25% and cell volume of +22% of normal cells. TEA caused a full Kv channel block, enhanced cell proliferation of about +30–40% and cell volume of 17%. These findings are in agreement with the fact that specific BK or Kv channel blockers are expected to increase cell volume and proliferation while specific channel openers are expected to reduce cell volume (Lang and Hoffmann, 2013).

In our experiments a significant cell proliferation was also observed in the presence of low concentrations of IbTX (10^−7^–10^−8^ M) that caused a partial reduction of the K^+^-current of about −10%. Currently, we can hypothesize that this effect may be unrelated to the conduction properties of the channel but rather may involve protein-protein interactions that lead to activation of intracellular signaling (Urrego et al., 2014). This finding appears to be in agreement with the idea that BKCa channels contribute to the high proliferative or invasive potential in a number of malignant cell lines (Weaver et al., 2006; Bloch et al., 2007; Ouadid-Ahidouch and Ahidouch, 2008; Sontheimer, 2008; Khaitan et al., 2009; Koehl et al., 2010). MaxiK channel over-expression has been correlated with the malignancy of human gliomas, which has been associated with an abnormal overactive gBKCa channel (Toro et al., 2014).

In HEK293 cells transfected with the recombinant channel subunits, the NS1619 prevented cell proliferation induced by the BKCa channel blockers reducing cell viability, in our experiments NS1619 enhanced proliferation of the SH-SY5Ycells (Chang et al., 2011; Tricarico et al., 2013). This apparent discrepancy can be explained, taking into account the different molecular composition and properties of the recombinant vs. the native BKCa channel subtypes functionally expressed in the cells. NS1619 may also exert unspecific actions, for instance against L-type Ca^2+^ channels (Park et al., 2007).

The cell proliferation induced by the IbTX and TEA was prevented by staurosporine suggesting that this phenomenon is mediated by PKC or other staurosporine-sensitive protein kinases. We tested this hypothesis investigating the PKC and PKA activities using an ELISA assay in the lysates of cells incubated for 6 h with BKCa and Kv channel modulators. We found a marked enhancement of the PKC activity in the cells following IbTX, NS1619, and TEA, while staurosporine significantly reduced the PKC activity without significantly affecting the PKA activity in the cell lysates.

In our experiments the involvement of the surface BKCa channel in cell proliferation was investigated by performing patch-clamp experiments and cell proliferation assays in the presence of IbTX which is a specific and almost impermeable BKCa channel blocker. The partial channel block and the proliferation induced by IbTX at a low concentration was accompanied by the enhancement of the number cells showing a normal morphology without the appearance of any abnormal cell population as determined by the cell volume assay performed in our experiments. The fact that IbTX at a 4 × 10^−7^ M concentration induced at comparable values of cell proliferation of +22 and +18% (diam. range: 13–16 μm) using the mitochondrial succinic dehydrogenase and the cell volume assays, respectively, suggested that the proliferative effect of this drug is mediated by a common target affecting either cell volume and the mitochondrial succinic dehydrogenase enzyme. Because IbTX is a relatively impermeant toxin, the main target of this action can be the surface BKCa channel. But it should be stressed that the contribution of the nuclear BKCa channels in the neuronal cell proliferation cannot be excluded (Li et al., 2014).

In conclusion, BKCa channel plays an essential role in the proliferation of the native human neuroblastoma cell line SH-SY5Y and this effect is mediated by PKC and PKA enzymes, other protein kinases sensitive to staurosporine may be also involved. Other than in cell proliferation, BKCa has been involved in cell migration. It should be stressed that BKCa channels are not currently considered an oncogene *per se*, but instead can modulate cell migration and invasion and act like a facilitator as it has been reported in glioma cells (Lisheng et al., 2014).

The BKCa/PKC/PKA pathway may play a role in the cell repair processes. PKC and PKA enzymes other than recognized pathways involved in the proliferative disorders (Parker et al., 2014), their induction/activation was recently associated with the cell repair processes. The PKC isoforms particularly the alpha type, regulates physiological processes such as phagocytosis, endocytosis and desmosome downregulation; exert a cytoprotecting role against some forms of tumors (Larsen et al., 2000; Cheeseman et al., 2006; Boyle et al., 2014; McHarg et al., 2014). These mechanisms promote cell plasticity function favoring cell survival. The induction/activation of the PKA subtypes is also involved in the cell cytoprotection in the neurite plasmalemma repair (Zuzek et al., 2013).

### Conflict of interest statement

The authors declare that the research was conducted in the absence of any commercial or financial relationships that could be construed as a potential conflict of interest.
